# Japanese Encephalitis: Estimating Future Trends in Asia

**DOI:** 10.3934/publichealth.2015.4.601

**Published:** 2015-08-31

**Authors:** Julia Metelka, Colin Robertson, Craig Stephen

**Affiliations:** 1 Department of Geography & Environmental Studies, Wilfrid Laurier University, Waterloo, ON, Canada; 2 Canadian Wildlife Health Cooperative, Saskatoon, Canada

**Keywords:** Japanese encephalitis, Asia, disease estimation, forecasting, population change, disease risk mapping

## Abstract

Limited surveillance programs and lack of diagnostic laboratory testing capacity in many low and middle income Asian countries have made it difficult to validate epidemiological patterns and anticipate future changes in disease risk. In this study, we consider the case of Japanese Encephalitis in Asia and examine how populations of human hosts and animal reservoirs are expected to change over the next three decades. Growth was modelled at the sub-national level for rural and urban areas to estimate where high-density, susceptible populations will potentially overlap with populations of the virus' amplifying host. High-risk areas based on these projections were compared to the current distribution of Japanese Encephalitis, and known immunization activities in order to identify areas of highest priority for concern. Results indicated that mapping JE risk factors at the sub-national level is an effective way to contextualize and supplement JE surveillance data. New patterns of risk factor change occurring in Southeast Asia were identified, including around major urban areas experiencing both urbanization and growth in pig populations. A hotspot analysis of pig-to-population ratio found a significant spatial cluster extending northward through Southeast Asia and interior China. Mapping forecasted changes in risk factors for JE highlights regions vulnerable to emerging zoonoses and may be an important tool for developing effecting transnational health policies.

## 1. Introduction

Japanese Encephalitis (JE) is the leading cause of viral encephalitis in Asia [1], [2]. It is estimated that approximately 67,900 cases occur annually [3]. The case fatality rate is 20–30% and 30–40% of survivors suffer from permanent neurological sequelae [3]. The disease is primarily acquired by children less than 15 years of age and is historically more common in rural and agricultural areas [1],[2],[3].

Confirmed cases of JE are reported to the World Health Organization through the programme for vaccine-preventable diseases (http://apps.who.int/immunization_monitoring/en/). Surveillance is absolutely necessary to guide immunization programs, target surveillance resources, set priorities, and can serve as an early warning system for identifying public health emergencies [4]. However, most human cases of JE are asymptomatic and go unreported, making the spatial distribution of the virus difficult to estimate.

The [4] estimates that approximately 1 in 250 of those who acquire the JE virus display encephalitis symptoms. For those that do experience disease, there is significant variation in health seeking behaviours across Asia, which distorts the sensitivity of disease surveillance systems. Factors such as the structure of the health care system (centralized or not), physical access to health care, propensity to adopt traditional and/or care services outside of the government system all vary across Asia and merge to obscure understanding of the disease burden due to JE. For example, [5] noted that many people in Nepal prefer traditional healers/medicine over hospitals, resulting in many unreported cases. [5] also mentioned that this preference for traditional healers was more prominent among poor and disadvantaged sectors of the population. There is therefore need to examine not only the reported cases of disease, but also the distribution of disease risk factors when estimating risk, developing health policy, and anticipating future changes.

There are several major contributing factors that lead to high-risk areas for JE. Firstly, landscape factors play an important role in vector-borne disease transmission. Increased area dedicated to land-use types that contribute to vector habitat promote virus transmission and risk to humans. Other landscape characteristics, such as fragmentation and heterogeneity, can also increase risk of vector-borne disease [6]. Secondly, susceptible populations mixed with pig populations are strongly linked to JE risk. Pigs are considered to be an amplifying host for the JE virus. If a pig is infected with the virus, the probability that there will be infected mosquitoes in their vicinity is significantly increased. Age is also linked to JE, as the virus is typically associated with disease in children for areas where it is endemic. However, where childhood immunization programs exist, risk is approximately equal for adults and children [7],[8].

The Morbidity and Mortality Weekly Report conducted by [3] summarized the status of JE surveillance programs in risk areas. The report concluded that as of 2012, 10 of 25 countries with JE risk conducted nation-wide surveillance programs. They reported that six countries do not conduct any form of surveillance. The true incidence of JE is largely unknown due to the lack of sufficient surveillance and diagnostic laboratory testing in many countries [2],[4].

Additional information is required to compensate for limitations in surveillance data to inform JE preparedness and response planning. In this study, we propose a method that compensates for under-reporting using demographic trends as a forecasting tool. We also estimate potential distribution of pig population density in Asia using livestock head count data from the United Nations' Food and Agricultural Organization (FAO). By estimating where people are in the near future, and where amplifying host are, we can identify areas where there may be more susceptible individuals and thus areas of higher risk for JE.

**Figure 1.**
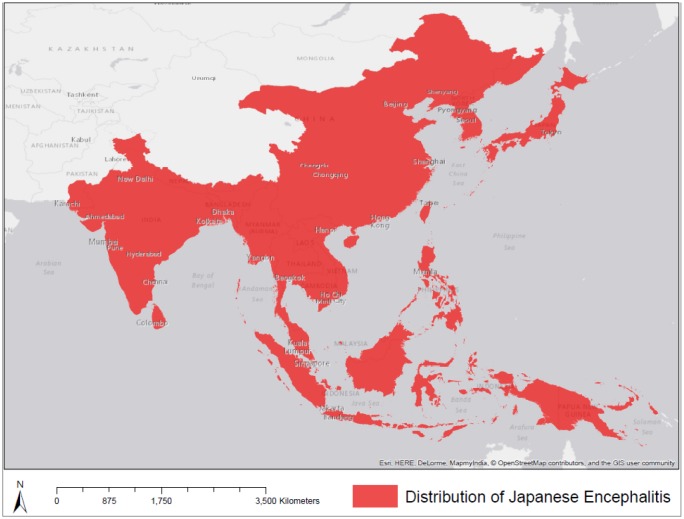
The current geographic distribution of Japanese Encephalitis.

## 2. Background

### 2.1. The Japanese Encephalitis Virus

The JE virus (JEV) is a Flavivirus transmitted via mosquitoes. The primary vector of JEV is from the genus *Culex*, although other mosquitoes are also known to carry the virus [9]. Rice agriculture areas provide ideal habitat for *Culex* mosquitoes, and thus risk of acquiring the virus tends to increase with proximity to paddy fields [10], [11]. [12] estimated that area dedicated to rice agriculture in Asia increased by 22% during the period 1963–2003, which has directly contributed to habitat expansion of *Culex* mosquitoes.

Wild birds and domesticated pigs are the primary hosts of the JEV. Wading birds from the family *Ardeidae* (herons, bitterns, and egrets) are a natural reservoir for the virus. These birds require shallow water bodies as feeding grounds [13] and thus frequent areas dedicated to rice agriculture. Species from the family *Suidae*, which include domesticated pigs and wild boar, are the main amplifying hosts of JEV [4],[10],[14]. When pigs are infected with JEV, they are able to infect many additional mosquitoes. Increasing area dedicated to rice agriculture and the growing pork industry in Asia presents a growing concern for JE transmission in the near future. The distribution of risk factors related to pig husbandry, mosquito life cycle development and habitat, and interactions between hosts and vectors is extremely difficult to track over large areas. As such, this paper focuses on human and pig population trends as a way to forecast future changes of JE in Asia.

### 2.2. The State of Japanese Encephalitis in Asia

JE was first identified in 1871 and the first recognized epidemic occurred in Japan in 1924 [15]. Since then, the geographic distribution of JE has been expanding [15]. [15] suggested that a changing landscape, involving deforestation and increased agricultural area (especially rice agriculture and pig farming), has promoted the spread of the disease. These landscape variables are associated with a population growth and change. Figure 1 displays the current geographic distribution of JE [2], [3].

### 2.3. Japanese Encephalitis in Urban Areas

[16] recently demonstrated how JE is becoming increasingly urban. For example, urbanized areas in the area of the National Capital Territory of Delhi, India provide sufficient breeding habitats for *Culex* mosquitoes that transmit the JE virus [16]. The presence of migratory *Ardeidae* birds and pig domestication in these urban settings has amplified JE transmission. [16] was the first to identify cases of JE in the Delhi area and also provided evidence that JE is becoming increasingly an urban, rather than just a rural issue.

[17] stated that rapid human population growth leading to crowding and poor sanitation has resulted in the proliferation of *Aedes* mosquitoes, which have the potential to carry the dengue virus (although this disease can be endemic within a human population without any animal reservoirs). These types of urban landscape characteristics have led to increased risk of vector-borne diseases in urban areas. It is for these reasons that this study eliminates differentiating between rural and urban area and risk of JE. We do however, differentiate between rural and urban population growth rates in Asia, as urbanization is an increasingly important process transforming exposure opportunities in Asia.

### 2.4. Land-use & Landcover Change

The population in Asia is expected to increase from 4.342 billion in 2012 to 5.164 billion by 2050 [18]. Land-use change and the expansion of agriculture, the key driving forces of JE transmission are expected to intensify as population increases [19]. [19] stated that during the period 1990-2008, agricultural land increased in area by over 8% in Southeast Asia. Pig-farming is also intensifying in Asia. [10] estimated that between 1990 and 2005, Cambodia, China, South Korea, Laos, Myanmar, Nepal, the Philippines, Sri Lanka, Thailand, and Vietnam all experienced increased pork production by between 12% and 381%. Such environmental factors, as well as population increases, will undoubtedly lead to an increased amount of JE cases unless otherwise managed.

### 2.5. Study Objectives

The objectives of this paper are to;

- Develop forecasts of populations vulnerable to JE across Asia for 2050
- Develop forecasts under high, medium and low scenarios of amplifying hosts for JE across Asia for 2050
- Identify locations where overlapping risk reservoirs, host populations, and facilitating contextual factors are present in 2050 given current knowledge which we aim to use to both present a methodology for anticipating future change in emerging diseases, and specific to JE, anticipating future patterns of risk for data-driven health policy formulation and disease management planning.

## 3. Materials and Method

### 3.1. Study Area

The study area includes all countries where JE has been identified. This includes most countries in South Asia, East Asia, and Southeast Asia as shown in Figure 1.

### 3.2. Data

Administrative boundaries were obtained from www.diva-gis.org [20] and were used for district-level analyses.

Two sets of population data were obtained. The first set was obtained from www.worldpop.org.uk and is presented as people per pixel for the year 2015 and is adjusted to match the UN Population Division estimates. The gridded population data set was use to estimate districts that were predominantly rural or urban, assuming that higher population densities occur in more urbanized areas.

The second population set was obtained from GeoHive at http://www.geohive.com/ [21]. Geo- Hive data were retrieved at one level below the national level for each country's most recent census and spatially joined to the administrative boundaries.

The United Nations World Urbanization Prospects project (UN WUP 2014) estimated rural and urban growth rates for five year intervals for each country (http://esa.un.org/unpd/wup/CD-ROM/). These growth rates were used to project district-level populations until the year 2050, this method will be discussed further in Section 3.4.

Two pig population datasets were obtained from the Food and Agriculture Organization of the United Nations (FAO). Yearly pig head counts for the period 1961–2012 were obtained for each country from the ‘Live Animals’ database [22]. Data from this database is only available at the country level and was not available for every country in the study area (Figure 1). A gridded livestock density map for pigs for the year 2005 was obtained from http://www.fao.org/ag/againfo/resources/en/glw/GLW_dens.html [23]. The livestock density raster was used to determine district-level pig populations, which was not available using 2013 data from the ‘Live Animals’ database.

Landcover data derived from MODIS satellite imagery was obtained from the Global Land Cover Facility (GLCF) at http://glcf.umd.edu/data/lc/. The GLCF classifies MODIS imagery into 16 landcover types at a 5 arc-minute (0.08333 degrees) spatial resolution [24]. These landcover types were used to define districts as being either rural or urban so that appropriate growth rates could be applied.

### 3.3. District Classification

[25] noted that “urban means nonagricultural”. Regions that exhibit high population densities may still focus on a rural way-of-life (i.e. agricultural activities), especially in Asia where many countries have rural populations of over 50% of the total population [26]. Therefore, to classify districts as being predominantly urban or rural, landcover characteristics from [24] were considered.

For a district to be classified as urban, two conditions were to be satisfied. The first condition required population densities to exceed 500 persons per square kilometre and that the majority pixel count was not ‘croplands’ as classified by [24]. The second condition stated that the majority pixel count was ‘Urban and built-up’ as classified by [24] within district boundaries.

**Figure 2.**
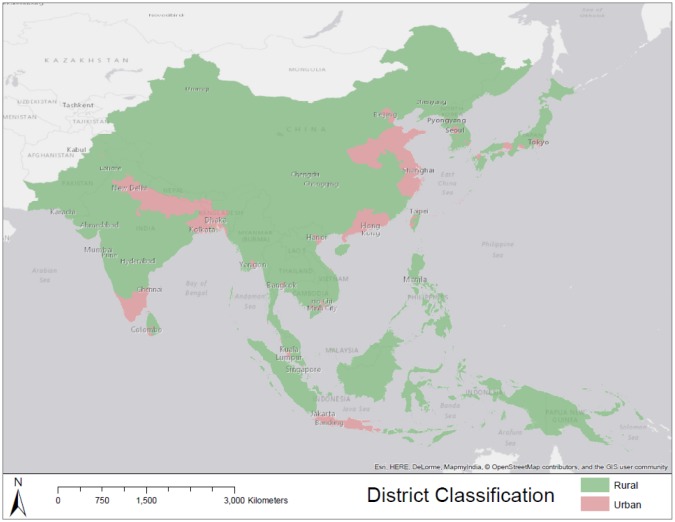
District classification as predominantly urban or predominantly rural.

For a district to be classified as being predominantly rural, it failed to meet the criteria of being defined as urban. Landcover characteristics were also considered, and if a district contained a majority of pixels classified as ‘croplands’ [24], then that district was classified as rural. Final district classification is shown in Figure 2.

### 3.4. Human Population Projections

The rate of urban population growth and rural population growth often differs. According to [27], the urban growth rate for South-central Asia and South-eastern for the period 1950-2000 was 3.34% and 4.02%, respectively. The rural population growth rates for these two regions were 1.84% and 1.53%, respectively [27]. For studies in population projection at a subnational level, it is thus necessary to specify rural and urban areas. However, the distinction is not always straightforward. In many Asian countries, a high population density may not always suggest that a region is dominated by an urban lifestyle. Many Asian countries are still characterized by a rural way of life, even though populations may be high [28]. In this study, we use the most recent rural and urban growth rates as determined by the [18] and apply them to districts classified in Section 3.3.

Five year average growth rates from WUP 2014 were used to project future population values. Population values were estimated for each 5 year interval's end year, which were then used to estimate the next interval's value. The geometric population growth model was used to estimate future populations as shown in Equation 1.

$$Pop_{F}=Pop_{C}{\left(1+\frac{r}{100}\right)}^{T}$$ (1)

Where *PopF* is the year for the estimated population, *PopC* is the value of the population for the districts most recent census, *r* is the population growth rate from UN WUP 2014, and *T* is the number of years between *PopF* and the country's last census year.

Based on the classification of each district being predominantly urban or rural, each country's most recent census and population growth rates from the UN's WUP 2014, population at the district level was calculated for the year 2050. We estimated that population in the study area increased from ≈ 3.9 billion for the year 2015 to ≈ 4.5 billion for the year 2050. Considering that the study area comprises most, but not all of Asia, these estimates are roughly in line with [18] estimates for the study area.

### 3.5. Pig Population Projections

Pig population data in raster format for the year 2005 were used to estimate district-level values. A zonal statistics summary was performed on this raster dataset and used to estimate pig population proportions at the district-level (i.e. zonal sum in district divided by country total). Tabular data from the FAO's ‘Live Animals’ database for each year for the period 1961–2013 at the country level were then multiplied by district-level proportions devised from the raster dataset.

Data for all countries in the study area were not available from the FAO's Live Animals database. Countries for which pig headcount data was obtained were; Bhutan, Brunei, Cambodia, China, Japan, Hong Kong, Macao SAR, Taiwan, North Korea, India, Indonesia, Laos, Malaysia, Myanmar, Papua New Guinea, South Korea, Sri Lanka, Thailand, Timor-Leste, and Vietnam.

At the country-level, pig livestock data from the FAO were used to estimate future values in low, medium, and high scenarios. Projections for the year 2050 were done using the ‘Forecast’ package in R, which is used for analyzing univariate time series data. Forecasts were calculated with a 75 percent prediction interval. The upper bounds, mean, and lower bounds of the prediction interval were used for high, medium, and low scenarios, respectively. The forecast function fits an exponential smoothing state (ETS) model to the time series data to predict future values [29]. Country-level pig projections were then allocated to district administrative boundaries according to their 2005 proportions.

### 3.6. Highly Vulnerable Regions

Highly vulnerable regions (HVR) for JE in 2050 were identified in two ways. Firstly we mapped the spatial overlap of population and pig populations. Population changes were categorized as either declining (% change < 0), stable (%change 0–5), low (%change 6–10), moderate (%change 7–20), and high (%change > 20). Pig populations were categorized using the same classification, and maps of growth classes were created and used to identify highly vulnerable regions.

Additionally, the percent changes in the ratio of pig populations to human populations were calculated and mapped. Spatial analysis of the percent change was used to derive hotspots of significant change using the local Getis-Ord spatial statistic. The local Getis-Ord statistic (Gi(d)) [30] takes a moving average of a numeric variable *x* within a spatial neighbourhood defined by a spatial weight matrix *w_ij_(d)* (where *d* is the maximum distance at which nearby points are included as neighbours) as:

$$G_{i}(d)=\frac{\sum_{j}w_{ij}(d)x_{j}}{\sum_{j=1}^{n}x_{j}}$$ (2)

over the total sum of *x*. In practice *Gi(d)* is defined as a standard normal deviate, and therefore can be interpreted as a z-score. We employ the *Gi(d)* statistic to identify significant spatial clusters where the relationship between pigs and people are expected to change.

Results of HVR mapping were interpreted relative to contextual variables relevant for JE risk, including landcover classification, vaccination status of populations, and the current distribution of JE incidence.

**Figure 3.**
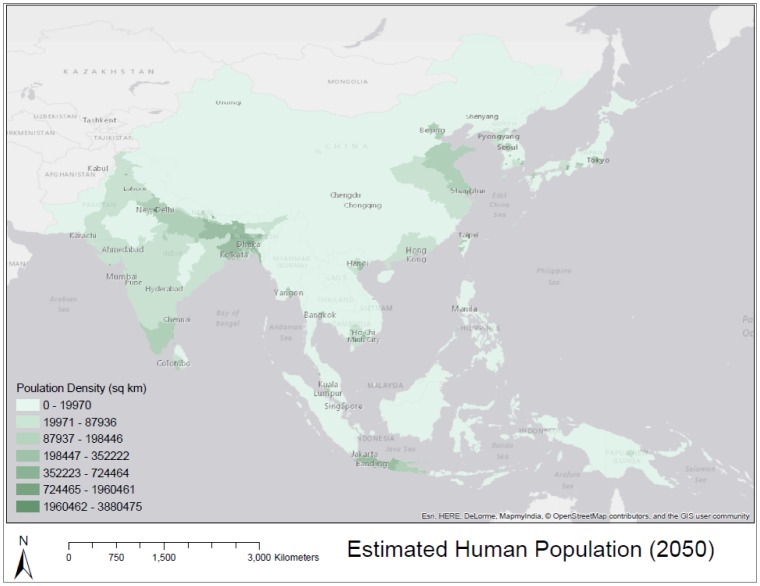
Estimated human population density for the year 2050.

**Figure 4.**
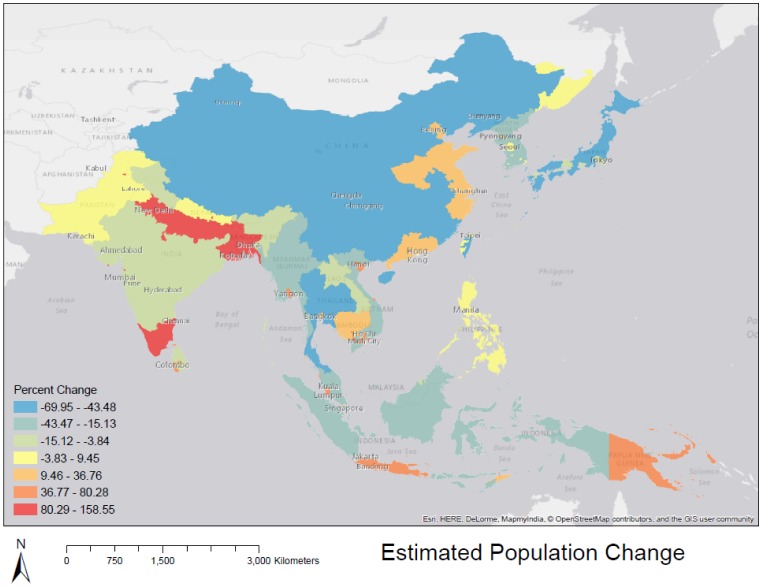
Estimated human population change at the district-level, Areas with higher rates of change are those classified as ‘urban’ in this study, and therefore experience higher rates of change.

## 4. Results

### 4.1. Human Population Change in Asia

Figure 3 shows our estimated population density, using urban/rural growth rate distinction, for the year 2050. Figure 4 shows our estimation of population change. While large regions of rural China are projected to experience population loss which dominate the map, urban areas in southeast Asia, and southern India are expected to experience positive change in population by 2050, reflecting the regional trend toward urbanization, described by the [18].

### 4.2. Pig Population Change in Asia

**Figure 5.**
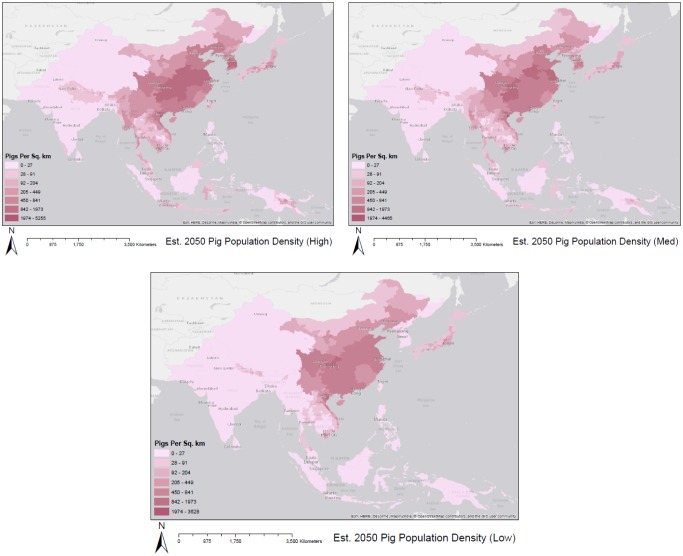
Estimated pig population density with high medium and low forecast scenarios for the year 2050.

Figure 5 displays our estimated pig projection and distribution at the district-level for the year 2050 under the three forecasted scenarios. Here, we see commonalities between the three forecasts. Central China, and pockets of Nepal, northern India, Japan, and North Korea exhibit high densities in all three scenarios.

### 4.3. Highly Vulnerable Regions in Asia

**Figure 6.**
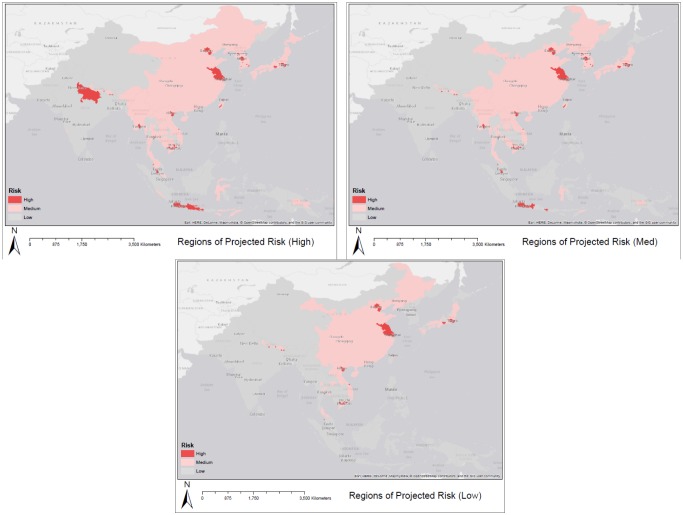
Regions with high pig densities (greater than 50/km^2^) and high human popu-lation densities (greater than 1000/km^2^) for the year 2050.

Maps of pig populations and human populations in 2050 were combined to identify regions that exhibit high human population densities (> 1 000 per sq. km) and high pig densities (> 50 per sq. km) for the year 2050 in Figure 6. Parts of eastern coastal China and urban regions in Southeast Asia are highlighted as particularly vulnerable in all three scenarios.

**Figure 7.**
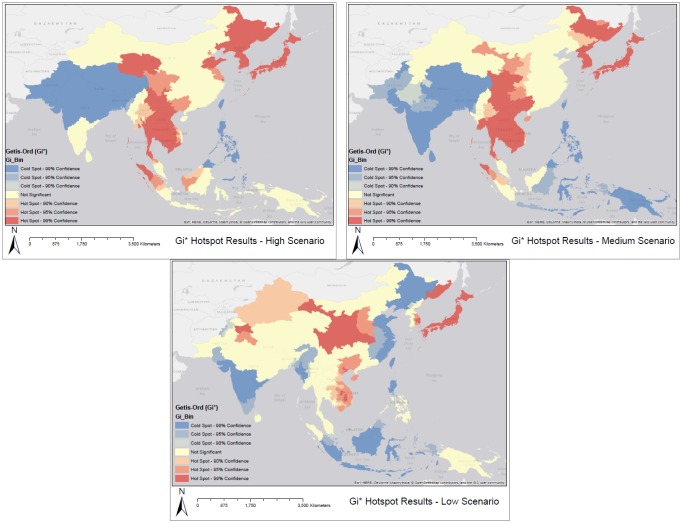
Gi* results. Red regions represent areas with high values of pigs per 100 000 people under the high medium and low forecasted scenarios for the year 2050.

The relationship between pigs and people is expressed in Figure 7 as the percentage change in the ratio of pigs to people. The Getis-Ord statistic highlights hotspots (significant increases) in pig:human populations and coldspots (significant decreases) based on the local Getis-Ord analysis.

## 5. Discussion

This study considers potential population trends and JEV amplifying host projections at the district-level in order to estimate regions of potential risk for acquiring Japanese Encephalitis. By projecting future spatial patterns of susceptible human populations and risk factors (pigs) we highlight regions that should be of special interest for disease intervention strategies.

**Figure 8.**
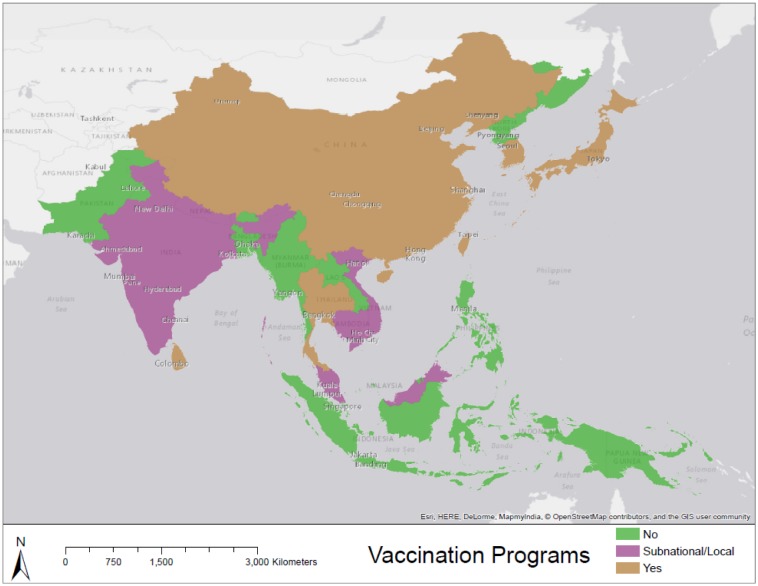
Country-level status of immunization programs. Regions depicted in green have no immunization strategies as of the year 2012 [3], [4].

Figure 6 displays regions where pig population densities and human population densities are expected to be high based on our estimations. It should be noted that these maps do not account for vaccination programs (Figure 8) and countries that are well-developed such as Japan may not be of _particular_ risk_._

Figure 7 highlights regions in which pig populations (under three different scenarios) and human populations are estimated to increase significantly by the year 2050. Figure 8 displays the status of immunization programs at the country level [34]. Regions depicted in green and pink either have no immunization program or a program that operates at sentinel sites. Many regions that are highlighted as high risk in Figure 7 and Figure 6 overlap with regions depicted in Figure 8 as having no immunization strategy. Because disease reporting is required for diseases at a national scale it is important to examine sub-national trends over large areas to reflect the underlying dynamics of the disease processes. Here we have focused on some key variables of interest in order to map patterns of risk and risk change over time.

However, the transmission cycle of JE is incredibly dynamic; it is dependent on the presence of vector breeding habit, agricultural practices, climate, and proximity of the population to agricultural areas as well as various social determinants. The number of JE cases within a country is also dependent on the presence of vaccination programs, the frequency and distribution of these programs within the country (i.e. located in rural vs. urban area), and effective surveillance. Future large-area mapping efforts may take into consideration some of the main JE drivers at a more granular level of spatial detail such as land-use change, paddy areas, population movements, and pig husbandry. Examples of this approach in Nepal [31],[32] have shed light on the regional distribution of risks, however obtaining detailed data for such variables over large areas at granular geographies remains a significant challenge.

Countries in Asia are also at different stages of development. [10] suggested that incidence of JE will increase mainly in low-income areas. Countries such as Cambodia, Laos, and Myanmar will likely see JE outbreaks in the near future due to increases in irrigated rice agriculture and pig rearing [10].

For example, during the period 1990-2005 Myanmar exhibited an increase of 47% in rice agriculture area as well as an increase of 381% in pork production [10]. [3] stated that Laos and Myanmar do not have JE immunization programs in place and that Cambodia only conducts surveillance in three of its twenty-three provinces. Considering these factors, we might expect that the incidence of JE may be greater in these countries than the current incidence as calculated by [2]. In contrast, incidence may decrease in regions where vaccination programs are in their early stages. Recent progress in JE prevention has caused increased awareness of the disease, funding, and the availability of improved vaccines, although only in more developed countries [3].

### 5.1. Limitations

The distinction between urban and rural districts is not always a dichotomy. [25] highlights the difficulties with this distinction. In this study, we choose to assign districts to one of these classes based off of landscape and population characteristics in order to project future populations at the district-level. The aim of this was to obtain fine-scale estimations of susceptible human populations.

This study is hindered by the quality of data used for analysis. We used the best available data to estimate potential trends and distributions of susceptible populations as well as the virus's host. Using livestock data for the period 1961–2013 to project future trends is not always straightforward and there is much uncertainty in these estimations. We have attempted to account for this uncertainty by forecasting pig populations under three scenarios using the upper and lower bounds of a prediction interval. Pig farming is also highly contingent on cultural factors that may not be represented in the data. For example, pig farming in Nepal has significantly increased after the year 1950 due to changing religious views and the tourism industry [33]. Thus, the data shows an upward trend that may stabilize in the near future. However, these relationships are incredibly difficult to map at such a large scale and are beyond the scope of this study. Also, we show how pig populations are expected to decline in India. This may not actually be the case and may be due to low-quality of data obtained by the FAO. It should be noted that pig population estimations were conducted to the best of our knowledge using the best data available.

Land-use and landcover change plays a prominent role in the transmission of vector-borne diseases. This study did not focus on land-use and landcover change as it was beyond the scope of this study. There are many small-scale characteristics that contribute to mosquito populations, which are not visible from large-scale remotely-sensed data such as [24]. For example, [34] found that certain species of *Culex* mosquitoes are abundant in urban areas of India and suggested that control measures should equally focus on urban and rural areas.

## 6. Conclusion

[4], [35] recommends that immunization is the most effective measure for preventing JE and should be extended to regions where JE is a public health problem. For many countries, resource constraints prevent the implementation immunization programs [3]. This study attempts to identify regions with high susceptible human populations and virus host populations. If regions with a high number of cases are identified, this may warrant increased attention for risk surveillance in this region.

This study examines disease risk factors at a large scale; however, many changes are occurring at a much smaller scale. For example, pig farming in Nepal is increasing due to the reduction of cultural biases against pigs [33] increasing the number of amplifying hosts in this region. It is evident that much work is needed at a smaller scale to fully understand the spatial dynamics of JE on a larger scale.

Pigs are involved in the transmission of other zoonotic diseases, such as *Streptococcus suis*, which can cause meningitis and other serious symptoms [36]. Using methods presented in this study, it is also possible to highlight regions where interventions on other such diseases should be focused.

Although this study highlights regions where there could be high increases in both human and pig populations, we do not discourage national-level interventions to prevent JE in other regions in Asia. We attempt to highlight regions that may be of importance in order to appropriate resources to combat JE in the case that resources are limited.

## References

[1] Mackenzie JS, Gubler DJ, Petersen LR (2004). Emerging Flaviviruses: The Spread and Resurgence of Japanese Encephalitis, West Nile and Dengue Viruses. Nature Med.

[2] Campbell GL, Hills SL, Fischer M, Jacobson JA, Hoke CH, Hombach JM (2011). Estimated Global Incidence of Japanese Encephalitis: A Systematic Review. Bulletin of the World Health Organization.

[3] Centers for Disease Control and Prevention (2013). Japanese encephalitis surveillance and immunization–Asia and the Western Pacific, 2012. MMWR Morbidity and mortality weekly report.

[4] World Health Organization (2014). Media Centre: Japanese Encephalitis.

[5] Akiba T, Osaka K, Tang S, Nakayama M, Yamamoto A, Kurane I (2001). Analysis of Japanese encephalitis epidemic in Western Nepal in 1997. Epidem infect.

[6] Lambin EF, Tran A, Vanwambeke SO, Linard C, Soti V (2010). Pathogenic Landscapes: Interactions Between Land, People, Disease Vectors, and Their Animal Hosts. Int J Health geogra.

[7] Wu YC, Huang YS, Chien LJ, Lin TL, Yueh YY, Tseng WL (1999). The epidemiology of Japanese encephalitis on Taiwan during 1966-1997. Am J tropica med hygie.

[8] Sohn YM (2000). Japanese encephalitis immunization in South Korea: past, present, and future. Emerg Infect Dis.

[9] Solomon T, Ni H, Beasley DW, Ekkelenkamp M, Cardosa MJ, Barrett AD (2003). Origin and Evolution of Japanese Encephalitis Virus in Southeast Asia. J Virol.

[10] Erlanger TE, Weiss S, Keiser J, Utzinger J, Wiedenmayer K (2009). Past, Present, and Future of Japanese Encephalitis. Emerg Infect Dis.

[11] Miller RH, Masuoka P, Klein TA, Kim HC, Somer T, Grieco J (2012). Ecological Niche Modeling to Es- timate the Distribution of Japanese Encephalitis Virus in Asia. PLoS Neglect Tropica Dis.

[12] Keiser J, Maltese MF, Erlanger TE, Bos R, Tanner M, Singer BH (2005). Effect of Irrigated Rice Agriculture on Japanese Encephalitis, Including Challenges and Opportunities for Integrated Vector Management. Acta Tropica.

[13] Sibley DA (2000). The Sibley Guide to Birds.

[14] Le Flohic G, Porphyre V, Barbazan P, Gonzalez JP (2013). Review of Climate, Landscape, and Viral Genetics as Drivers of the Japanese Encephalitis Virus Ecology. PLoS Neglect Tropica Dis.

[15] Misra UK, Kalita J (2010). Overview: Japanese Encephalitis. P Neurobiology.

[16] Kumari R, Kumar K, Rawat A, Singh G, Yadav NK, Chauhan LS (2013). First indigenous transmission of Japanese Encephalitis in urban areas of National Capital Territory of Delhi, India. Tropica Med & Inter Health.

[17] Esteva L, Vargas C (1999). A model for dengue disease with variable human population. J Mathem Bio.

[18] Department of Economic and Social Affiars: Population Division of the United Nations (2014). The World Population Situation in 2014.

[19] Coker RJ, Hunter BM, Rudge JW (2011). Emerging Infectious Diseases in Southeast Asia: Regional Challenges to Control. The Lancet.

[20] DIVA-GIS (2013). Country Level Data.

[21] GeoHive (2013). GeoHive Historic, Current, and Future Population of Asia.

[22] Food and Agriculture Organization of the United Nations (2014). Production/Live Animals. United Nations.

[23] Animal Production and Health: Food and Agriculture Organization of the United Nations Gridded Livestock of the World (GLW).

[24] Channan S, Collins K, Emanuel WR (2014). Global mosaics of the standard MODIS land cover type data.

[25] Weeks JR (2010). Defining urban areas. Remote Sensing of Urban and Suburban Areas.

[26] The World Bank (2013). Agriculture & Rural Development: Rural Population (% of total population).

[27] United Nations (2002). World Urbanization Prospects: The 2001 Revision.

[28] Tacoli C (1998). Rural-urban interactions; a guide to the literature. Environment and Urbanization.

[29] Yeasmin K, Hyndman R (2008). Automatic Time Series Forecasting: The forecast Package for R. Journal of Statistical Software.

[30] Getis A, Ord JK (1992). The analysis of spatial association by use of distance statistics. Geographi Analy.

[31] Impoinvil DE, Solomon T, Schluter WW, Rayamajhi A, Bichha RP, Shakya G (2011). The Spatial Heterogeneity Between Japanese Encephalitis Incidence Distribution and Environmental Variables in Nepal. PloS one.

[32] Robertson C, Pant DK, Joshi DD, Sharma M, Dahal M, Stephen C (2013). Comparative Spatial Dynamics of Japanese Encephalitis and Acute Encephalitis Syndrome in Nepal. PloS one.

[33] Dhakal S, Joshi DD, Ale A, Sharma M, Dahal M, Shah Y (2014). Regional Variation in Pig Farmer Awareness and Actions Regarding Japanese Encephalitis in Nepal: Implications for Public Health Education. PloS one.

[34] Murty US, Rao MS, Arunachalam N (2010). The effects of climatic factors on the distribution and abundance of Japanese encephalitis vectors in Kurnool district of Andhra Pradesh, India. J Vector Borne Dis.

[35] The World Health Organization (2008). WHO - Recommended Standards for Surveillance of Selected Vaccine-Preventable Diseases.

[36] Gottschalk M, Segura M (2000). The pathogenesis of the meningitis caused by Streptococcus suis: the unresolved questions. Veterin microbio.

